# (2-Oxo-2*H*-benzo[*h*]chromen-4-yl)methyl morpholine-4-carbodithio­ate

**DOI:** 10.1107/S160053681201094X

**Published:** 2012-03-17

**Authors:** Rajni Kant, Vivek K. Gupta, Kamini Kapoor, Gurvinder Kour, K. Mahesh Kumar, N. M. Mahabaleshwaraiah, O. Kotresh

**Affiliations:** a X-ray Crystallography Laboratory, Post-Graduate Department of Physics & Electronics, University of Jammu, Jammu Tawi 180 006, India; b Department of Chemistry, Karnatak Science College, Dharwad 580 001, Karnataka, India

## Abstract

In the title compound, C_19_H_17_NO_3_S_2_, the morpholine ring is in a chair conformation. In the coumarin ring system, the dihedral angle between the benzene and pyran rings is 3.9 (1)°. In the crystal, weak C—H⋯O inter­actions link the mol­ecules into corrugated layers parallel to (102). The crystal packing also exhibits π–π inter­actions, with distances of 3.644 (1) and 3.677 (1) Å between the centroids of the benzene rings of neighbouring mol­ecules.

## Related literature

For the biological activity of coumarins, see: Kontogiorgis & Hadjipavlou-Litina (2004 ▶). For a related structure, see: Kumar *et al.* (2012 ▶). For standard bond lengths, see: Allen *et al.* (1987 ▶).
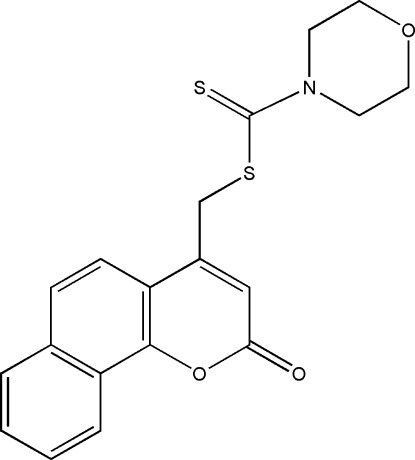


## Experimental

### 

#### Crystal data

C_19_H_17_NO_3_S_2_
*M* *_r_* = 371.46
Monoclinic, 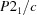

*a* = 13.0928 (4) Å
*b* = 11.6978 (3) Å
*c* = 11.3673 (3) Å
β = 99.232 (3)°
*V* = 1718.43 (8) Å^3^
*Z* = 4
Mo *K*α radiation
μ = 0.33 mm^−1^
*T* = 293 K
0.3 × 0.2 × 0.1 mm

#### Data collection

Oxford Diffraction Xcalibur Sapphire3 diffractometer
Absorption correction: multi-scan (*CrysAlis RED*; Oxford Diffraction, 2010 ▶) *T*_min_ = 0.818, *T*_max_ = 1.000
18655 measured reflections
3017 independent reflections
2457 reflections with *I* > 2σ(*I*)
*R*_int_ = 0.034

#### Refinement

*R*[*F* ^2^ > 2σ(*F* ^2^)] = 0.038
*wR*(*F* ^2^) = 0.104
*S* = 1.05
3017 reflections
226 parameters
H-atom parameters constrained
Δρ_max_ = 0.32 e Å^−3^
Δρ_min_ = −0.19 e Å^−3^

### 

Data collection: *CrysAlis PRO* (Oxford Diffraction, 2010 ▶); cell refinement: *CrysAlis PRO*; data reduction: *CrysAlis RED* (Oxford Diffraction, 2010 ▶); program(s) used to solve structure: *SHELXS97* (Sheldrick, 2008 ▶); program(s) used to refine structure: *SHELXL97* (Sheldrick, 2008 ▶); molecular graphics: *ORTEP-3* (Farrugia, 1997 ▶); software used to prepare material for publication: *PLATON* (Spek, 2009 ▶).

## Supplementary Material

Crystal structure: contains datablock(s) I, global. DOI: 10.1107/S160053681201094X/cv5260sup1.cif

Structure factors: contains datablock(s) I. DOI: 10.1107/S160053681201094X/cv5260Isup2.hkl

Supplementary material file. DOI: 10.1107/S160053681201094X/cv5260Isup3.cml

Additional supplementary materials:  crystallographic information; 3D view; checkCIF report

## Figures and Tables

**Table 1:** Hydrogen-bond geometry (Å, °)

*D*—H⋯*A*	*D*—H	H⋯*A*	*D*⋯*A*	*D*—H⋯*A*
C9—H9⋯O3^i^	0.93	2.42	3.346 (3)	173
C18—H18*A*⋯O2^ii^	0.97	2.56	3.466 (3)	155

Symmetry codes: (i) 
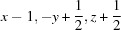
; (ii) 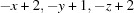.

**Fig. 1.**
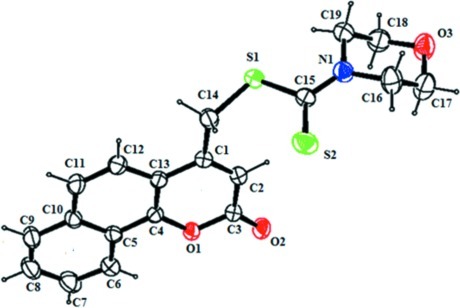
The molecular structure of the title compound. Displacement ellipsoids are drawn at the 40% probability level. H atoms are shown as small spheres of arbitrary radii.

## supplementary crystallographic information

### Comment

Coumarins (2H-1-benzopyran-2-ones) form a distinct class of oxygen containing heterocycles and are widely distributed in nature. Coumarins represent a class of naturally and synthetically obtained compounds that possess a wide variety of biological activities. Specifically coumarins are reported to possess antiallergic, anticoagulant, antidiabetic activities and analgesic properties (Kontogiorgis & Hadjipavlou-Litina, 2004). In continuation of our interest on crystal structures study of coumarin derivatives (Kumar *et al.*, 2012), we report the crystal structure of the title compound (I).

In (I) (Fig. 1), all bond lengths and angles are within normal ranges (Allen *et al.*, 1987) and are in a good agreement with those in related structure (Kumar *et al.*, 2012). The morpholine ring adopts a chair conformation. The dihedral angle bewteen the pyran and benzene rings in the coumarin fragment is 3.9 (1)°. Weak intermolecular C—H···O interactions (Table 1)link the molecules into corrugated layers parallel to (102) plane. The crystal packing exhibits π-π stacking interactions. The first of these is between the benzene ring C4/C5/C10-C13 and its symmetry-related partner at (1-x, 1-y, -z) with a distance of 3.644 (1) Å between the ring centroids. Another π-π interaction is between the benzene ring C4/C5/C10-C13 and the benzene ring C5-C10 at (1-x, 1-y,-z) with a distance of 3.677 (1) Å between the ring centroids.

### Experimental

A mixture of 2.73g (0.01 mol) of 7,8-benzo- 4-bromomethyl coumarin and 2.00g (0.01 mol) of potassium salt of morpholine-1-dithiocarbomate in 30 ml dry alcohol was stirrer for 12 hours at room temperature (the reaction was monitored by TLC). The solvent was evaporated and the solid was extracted twice with MDC –water mixture. The organic solvent was dried over CaCl2, evaporated the solvent and recrystallised from ethanol-chloroform. A slow evaporation technique was used to grow crystals suitable for diffraction studies in an ethanol-chloroform mixture. Yield=89%, m.p.-182-84oC. IR(KBr): 1717cm-1(C=O), 1423.8cm-1 (C=S), 849cm-1(C-N), 1111.7 (C-O-C).GCMS: m/e: 371.06. 1H- NMR(300MHz, CdCl3,δppm):2.81(s, 4H, C13 & C17-H), 1.73(s, 8H, C16,C17, C18 & C19-H), 4.32 (s,2H, C4-CH2), 7.26 (s,1H, C2-H), 7.45 (d,1H, C12-H), 7.63 (t,1H, C11-H), 7.66 (t,1H, C8-H), 7.91 (d,1H, C7-H), 7.97 (d,1H, C9-H), 8.47 (d,1H C6-H).Elemental analysis: C, 61.40; H, 4.56; N, 3.73; O, 12.90; S, 16.8 M.P.:

### Refinement

All H atoms were positioned geometrically and were treated as riding on their parent C atoms, with C—H distances of 0.93–0.97 Å and with *U*_iso_(H) = 1.2U_eq_(C).

### Crystal data

**Table:**

C_19_H_17_NO_3_S_2_	*F*(000) = 776
*M**_r_* = 371.46	*D*_x_ = 1.436 Mg m^−^^3^
Monoclinic, *P*2_1_/*c*	Mo *K*α radiation, λ = 0.71073 Å
Hall symbol: -P 2ybc	Cell parameters from 9000 reflections
*a* = 13.0928 (4) Å	θ = 3.5–29.0°
*b* = 11.6978 (3) Å	µ = 0.33 mm^−^^1^
*c* = 11.3673 (3) Å	*T* = 293 K
β = 99.232 (3)°	Plate shaped, light yellow
*V* = 1718.43 (8) Å^3^	0.3 × 0.2 × 0.1 mm
*Z* = 4	

### Data collection

**Table:**

Oxford Diffraction Xcalibur Sapphire3 diffractometer	3017 independent reflections
Radiation source: fine-focus sealed tube	2457 reflections with *I* > 2σ(*I*)
Graphite monochromator	*R*_int_ = 0.034
Detector resolution: 16.1049 pixels mm^-1^	θ_max_ = 25.0°, θ_min_ = 3.5°
ω scans	*h* = −15→15
Absorption correction: multi-scan (*CrysAlis RED*; Oxford Diffraction, 2010)	*k* = −13→13
*T*_min_ = 0.818, *T*_max_ = 1.000	*l* = −13→13
18655 measured reflections	

### Refinement

**Table:**

Refinement on *F*^2^	Primary atom site location: structure-invariant direct methods
Least-squares matrix: full	Secondary atom site location: difference Fourier map
*R*[*F*^2^ > 2σ(*F*^2^)] = 0.038	Hydrogen site location: inferred from neighbouring sites
*wR*(*F*^2^) = 0.104	H-atom parameters constrained
*S* = 1.05	*w* = 1/[σ^2^(*F*_o_^2^) + (0.0471*P*)^2^ + 0.803*P*] where *P* = (*F*_o_^2^ + 2*F*_c_^2^)/3
3017 reflections	(Δ/σ)_max_ = 0.001
226 parameters	Δρ_max_ = 0.32 e Å^−^^3^
0 restraints	Δρ_min_ = −0.19 e Å^−^^3^

### Special details

**Table:**

Experimental. CrysAlisPro, Oxford Diffraction Ltd., Version 1.171.34.40 (release 27-08-2010 CrysAlis171 .NET) (compiled Aug 27 2010,11:50:40) Empirical absorption correction using spherical harmonics, implemented in SCALE3 ABSPACK scaling algorithm.
Geometry. All esds (except the esd in the dihedral angle between two l.s. planes) are estimated using the full covariance matrix. The cell esds are taken into account individually in the estimation of esds in distances, angles and torsion angles; correlations between esds in cell parameters are only used when they are defined by crystal symmetry. An approximate (isotropic) treatment of cell esds is used for estimating esds involving l.s. planes.
Refinement. Refinement of F^2^ against ALL reflections. The weighted R-factor wR and goodness of fit S are based on F^2^, conventional R-factors R are based on F, with F set to zero for negative F^2^. The threshold expression of F^2^ > 2sigma(F^2^) is used only for calculating R-factors(gt) etc. and is not relevant to the choice of reflections for refinement. R-factors based on F^2^ are statistically about twice as large as those based on F, and R- factors based on ALL data will be even larger.

### Fractional atomic coordinates and isotropic or equivalent isotropic displacement parameters (Å^2^)

**Table:**

	*x*	*y*	*z*	*U*_iso_*/*U*_eq_	
S1	0.85592 (4)	0.20784 (5)	0.78872 (5)	0.04418 (18)	
S2	0.82222 (5)	0.40595 (6)	0.61667 (6)	0.0577 (2)	
O1	0.74719 (10)	0.50873 (12)	1.10256 (12)	0.0393 (3)	
O2	0.90505 (12)	0.56115 (14)	1.08171 (15)	0.0528 (4)	
O3	1.20360 (13)	0.26933 (17)	0.65798 (18)	0.0683 (5)	
N1	0.98691 (14)	0.26938 (16)	0.65122 (16)	0.0461 (5)	
C1	0.74297 (15)	0.35148 (17)	0.91788 (17)	0.0359 (5)	
C2	0.82708 (16)	0.41668 (18)	0.95188 (18)	0.0399 (5)	
H2	0.8838	0.4079	0.9128	0.048*	
C3	0.83295 (16)	0.49940 (18)	1.04603 (19)	0.0392 (5)	
C4	0.66310 (15)	0.43868 (17)	1.07426 (18)	0.0342 (4)	
C5	0.58582 (15)	0.45029 (18)	1.14826 (18)	0.0375 (5)	
C6	0.59575 (18)	0.5245 (2)	1.2473 (2)	0.0478 (6)	
H6	0.6539	0.5708	1.2654	0.057*	
C7	0.5195 (2)	0.5279 (2)	1.3166 (2)	0.0594 (7)	
H7	0.5270	0.5758	1.3828	0.071*	
C8	0.4307 (2)	0.4611 (2)	1.2901 (2)	0.0594 (7)	
H8	0.3795	0.4654	1.3379	0.071*	
C9	0.41879 (17)	0.3897 (2)	1.1947 (2)	0.0523 (6)	
H9	0.3592	0.3456	1.1776	0.063*	
C10	0.49592 (15)	0.38159 (18)	1.12088 (19)	0.0405 (5)	
C11	0.48607 (16)	0.30730 (19)	1.0208 (2)	0.0460 (5)	
H11	0.4257	0.2650	1.0002	0.055*	
C12	0.56292 (16)	0.29700 (19)	0.9549 (2)	0.0426 (5)	
H12	0.5549	0.2463	0.8910	0.051*	
C13	0.65541 (15)	0.36174 (17)	0.98100 (18)	0.0346 (4)	
C14	0.73585 (16)	0.2666 (2)	0.8168 (2)	0.0455 (5)	
H14A	0.7022	0.3037	0.7445	0.055*	
H14B	0.6916	0.2040	0.8334	0.055*	
C15	0.89538 (16)	0.29882 (18)	0.67899 (18)	0.0389 (5)	
C16	1.03585 (19)	0.3310 (3)	0.5620 (2)	0.0592 (7)	
H16A	0.9953	0.3981	0.5349	0.071*	
H16B	1.0386	0.2820	0.4937	0.071*	
C17	1.1420 (2)	0.3656 (3)	0.6158 (3)	0.0657 (7)	
H17A	1.1743	0.4058	0.5568	0.079*	
H17B	1.1385	0.4175	0.6815	0.079*	
C18	1.15784 (19)	0.2117 (2)	0.7477 (2)	0.0595 (7)	
H18A	1.1555	0.2632	0.8142	0.071*	
H18B	1.2004	0.1466	0.7770	0.071*	
C19	1.05149 (17)	0.1718 (2)	0.7007 (2)	0.0521 (6)	
H19A	1.0540	0.1152	0.6389	0.063*	
H19B	1.0213	0.1364	0.7642	0.063*	

### Atomic displacement parameters (Å^2^)

**Table:**

	*U*^11^	*U*^22^	*U*^33^	*U*^12^	*U*^13^	*U*^23^
S1	0.0474 (3)	0.0374 (3)	0.0509 (3)	0.0035 (2)	0.0176 (3)	−0.0012 (2)
S2	0.0595 (4)	0.0539 (4)	0.0601 (4)	0.0195 (3)	0.0114 (3)	0.0097 (3)
O1	0.0370 (8)	0.0371 (8)	0.0459 (8)	−0.0050 (6)	0.0130 (6)	−0.0069 (6)
O2	0.0448 (9)	0.0505 (10)	0.0661 (11)	−0.0162 (8)	0.0178 (8)	−0.0156 (8)
O3	0.0451 (10)	0.0716 (13)	0.0908 (13)	0.0030 (9)	0.0190 (9)	0.0225 (11)
N1	0.0415 (10)	0.0494 (11)	0.0487 (11)	0.0067 (9)	0.0109 (8)	0.0093 (9)
C1	0.0365 (11)	0.0320 (11)	0.0400 (11)	0.0015 (9)	0.0087 (9)	−0.0001 (9)
C2	0.0394 (12)	0.0378 (12)	0.0455 (12)	−0.0019 (10)	0.0159 (9)	−0.0023 (9)
C3	0.0372 (11)	0.0357 (11)	0.0466 (12)	−0.0024 (10)	0.0124 (9)	0.0001 (9)
C4	0.0310 (10)	0.0299 (10)	0.0423 (11)	0.0002 (8)	0.0074 (9)	0.0047 (9)
C5	0.0363 (11)	0.0339 (11)	0.0434 (11)	0.0075 (9)	0.0101 (9)	0.0071 (9)
C6	0.0471 (13)	0.0464 (13)	0.0523 (13)	0.0020 (11)	0.0157 (11)	−0.0034 (11)
C7	0.0645 (17)	0.0640 (17)	0.0547 (15)	0.0105 (14)	0.0241 (12)	−0.0039 (12)
C8	0.0524 (15)	0.0662 (17)	0.0668 (17)	0.0141 (13)	0.0317 (13)	0.0105 (14)
C9	0.0368 (12)	0.0556 (15)	0.0679 (16)	0.0036 (11)	0.0184 (11)	0.0114 (13)
C10	0.0320 (11)	0.0395 (12)	0.0513 (13)	0.0073 (9)	0.0104 (9)	0.0111 (10)
C11	0.0314 (11)	0.0433 (13)	0.0631 (15)	−0.0039 (10)	0.0066 (10)	0.0032 (11)
C12	0.0373 (11)	0.0397 (12)	0.0505 (12)	−0.0032 (10)	0.0059 (9)	−0.0035 (10)
C13	0.0313 (10)	0.0315 (10)	0.0412 (11)	0.0013 (9)	0.0062 (8)	0.0034 (9)
C14	0.0392 (12)	0.0473 (13)	0.0521 (13)	−0.0058 (10)	0.0142 (10)	−0.0126 (10)
C15	0.0411 (12)	0.0394 (12)	0.0365 (11)	0.0011 (9)	0.0076 (9)	−0.0043 (9)
C16	0.0541 (15)	0.0763 (18)	0.0482 (14)	0.0025 (13)	0.0117 (11)	0.0168 (13)
C17	0.0595 (16)	0.0668 (18)	0.0714 (17)	−0.0074 (14)	0.0129 (13)	0.0187 (14)
C18	0.0513 (15)	0.0550 (16)	0.0717 (16)	0.0093 (12)	0.0079 (12)	0.0117 (13)
C19	0.0487 (14)	0.0452 (14)	0.0657 (15)	0.0080 (11)	0.0193 (12)	0.0051 (11)

### Geometric parameters (Å, º)

**Table:**

S1—C15	1.778 (2)	C7—H7	0.9300
S1—C14	1.791 (2)	C8—C9	1.358 (4)
S2—C15	1.665 (2)	C8—H8	0.9300
O1—C4	1.369 (2)	C9—C10	1.416 (3)
O1—C3	1.385 (2)	C9—H9	0.9300
O2—C3	1.206 (2)	C10—C11	1.421 (3)
O3—C17	1.422 (3)	C11—C12	1.353 (3)
O3—C18	1.432 (3)	C11—H11	0.9300
N1—C15	1.333 (3)	C12—C13	1.419 (3)
N1—C16	1.472 (3)	C12—H12	0.9300
N1—C19	1.477 (3)	C14—H14A	0.9700
C1—C2	1.344 (3)	C14—H14B	0.9700
C1—C13	1.452 (3)	C16—C17	1.482 (3)
C1—C14	1.511 (3)	C16—H16A	0.9700
C2—C3	1.436 (3)	C16—H16B	0.9700
C2—H2	0.9300	C17—H17A	0.9700
C4—C13	1.382 (3)	C17—H17B	0.9700
C4—C5	1.422 (3)	C18—C19	1.484 (3)
C5—C6	1.411 (3)	C18—H18A	0.9700
C5—C10	1.418 (3)	C18—H18B	0.9700
C6—C7	1.369 (3)	C19—H19A	0.9700
C6—H6	0.9300	C19—H19B	0.9700
C7—C8	1.392 (4)		
			
C15—S1—C14	104.92 (11)	C11—C12—C13	121.5 (2)
C4—O1—C3	121.69 (16)	C11—C12—H12	119.3
C17—O3—C18	109.50 (18)	C13—C12—H12	119.3
C15—N1—C16	122.97 (19)	C4—C13—C12	117.56 (18)
C15—N1—C19	126.25 (18)	C4—C13—C1	117.83 (18)
C16—N1—C19	110.76 (18)	C12—C13—C1	124.60 (19)
C2—C1—C13	119.11 (19)	C1—C14—S1	116.03 (15)
C2—C1—C14	122.65 (18)	C1—C14—H14A	108.3
C13—C1—C14	118.24 (18)	S1—C14—H14A	108.3
C1—C2—C3	122.69 (19)	C1—C14—H14B	108.3
C1—C2—H2	118.7	S1—C14—H14B	108.3
C3—C2—H2	118.7	H14A—C14—H14B	107.4
O2—C3—O1	116.48 (19)	N1—C15—S2	124.86 (16)
O2—C3—C2	126.85 (19)	N1—C15—S1	112.65 (15)
O1—C3—C2	116.66 (18)	S2—C15—S1	122.47 (12)
O1—C4—C13	121.80 (17)	N1—C16—C17	109.4 (2)
O1—C4—C5	115.18 (18)	N1—C16—H16A	109.8
C13—C4—C5	123.01 (19)	C17—C16—H16A	109.8
C6—C5—C10	119.38 (19)	N1—C16—H16B	109.8
C6—C5—C4	123.2 (2)	C17—C16—H16B	109.8
C10—C5—C4	117.41 (19)	H16A—C16—H16B	108.2
C7—C6—C5	119.7 (2)	O3—C17—C16	111.5 (2)
C7—C6—H6	120.2	O3—C17—H17A	109.3
C5—C6—H6	120.2	C16—C17—H17A	109.3
C6—C7—C8	121.3 (2)	O3—C17—H17B	109.3
C6—C7—H7	119.4	C16—C17—H17B	109.3
C8—C7—H7	119.4	H17A—C17—H17B	108.0
C9—C8—C7	120.2 (2)	O3—C18—C19	111.5 (2)
C9—C8—H8	119.9	O3—C18—H18A	109.3
C7—C8—H8	119.9	C19—C18—H18A	109.3
C8—C9—C10	120.8 (2)	O3—C18—H18B	109.3
C8—C9—H9	119.6	C19—C18—H18B	109.3
C10—C9—H9	119.6	H18A—C18—H18B	108.0
C9—C10—C5	118.6 (2)	N1—C19—C18	110.0 (2)
C9—C10—C11	122.2 (2)	N1—C19—H19A	109.7
C5—C10—C11	119.20 (19)	C18—C19—H19A	109.7
C12—C11—C10	121.2 (2)	N1—C19—H19B	109.7
C12—C11—H11	119.4	C18—C19—H19B	109.7
C10—C11—H11	119.4	H19A—C19—H19B	108.2
			
C13—C1—C2—C3	−2.1 (3)	C5—C4—C13—C12	3.8 (3)
C14—C1—C2—C3	178.4 (2)	O1—C4—C13—C1	4.1 (3)
C4—O1—C3—O2	−176.30 (18)	C5—C4—C13—C1	−175.22 (18)
C4—O1—C3—C2	3.2 (3)	C11—C12—C13—C4	−2.0 (3)
C1—C2—C3—O2	−179.9 (2)	C11—C12—C13—C1	177.0 (2)
C1—C2—C3—O1	0.7 (3)	C2—C1—C13—C4	−0.2 (3)
C3—O1—C4—C13	−5.7 (3)	C14—C1—C13—C4	179.26 (19)
C3—O1—C4—C5	173.65 (17)	C2—C1—C13—C12	−179.2 (2)
O1—C4—C5—C6	−2.7 (3)	C14—C1—C13—C12	0.3 (3)
C13—C4—C5—C6	176.6 (2)	C2—C1—C14—S1	27.7 (3)
O1—C4—C5—C10	178.51 (17)	C13—C1—C14—S1	−151.80 (16)
C13—C4—C5—C10	−2.2 (3)	C15—S1—C14—C1	−92.49 (18)
C10—C5—C6—C7	1.0 (3)	C16—N1—C15—S2	1.3 (3)
C4—C5—C6—C7	−177.8 (2)	C19—N1—C15—S2	−177.02 (18)
C5—C6—C7—C8	−1.3 (4)	C16—N1—C15—S1	179.49 (18)
C6—C7—C8—C9	0.7 (4)	C19—N1—C15—S1	1.2 (3)
C7—C8—C9—C10	0.2 (4)	C14—S1—C15—N1	177.39 (16)
C8—C9—C10—C5	−0.4 (3)	C14—S1—C15—S2	−4.34 (16)
C8—C9—C10—C11	179.6 (2)	C15—N1—C16—C17	126.5 (2)
C6—C5—C10—C9	−0.1 (3)	C19—N1—C16—C17	−55.0 (3)
C4—C5—C10—C9	178.67 (19)	C18—O3—C17—C16	−60.7 (3)
C6—C5—C10—C11	179.8 (2)	N1—C16—C17—O3	58.7 (3)
C4—C5—C10—C11	−1.4 (3)	C17—O3—C18—C19	59.6 (3)
C9—C10—C11—C12	−176.9 (2)	C15—N1—C19—C18	−127.2 (2)
C5—C10—C11—C12	3.2 (3)	C16—N1—C19—C18	54.3 (3)
C10—C11—C12—C13	−1.5 (3)	O3—C18—C19—N1	−56.7 (3)
O1—C4—C13—C12	−176.88 (18)		

### Hydrogen-bond geometry (Å, º)

**Table:**

*D*—H···*A*	*D*—H	H···*A*	*D*···*A*	*D*—H···*A*
C9—H9···O3^i^	0.93	2.42	3.346 (3)	173
C18—H18*A*···O2^ii^	0.97	2.56	3.466 (3)	155

Symmetry codes: (i) *x*−1, −*y*+1/2, *z*+1/2; (ii) −*x*+2, −*y*+1, −*z*+2.
